# Color-Modulated *Spirulina* Biomass Produced Under Heterotrophic Cultivation as a Sustainable Food Ingredient

**DOI:** 10.3390/foods15152761

**Published:** 2026-08-06

**Authors:** Gabriela Moura Kurowiski de Brito, Jorge Alberto Vieira Costa, Luísa Barreira, Michele Greque de Morais

**Affiliations:** 1 Laboratory of Microbiology and Biochemistry, College of Chemistry and Food Engineering, Federal University of Rio Grande, Rio Grande 96203-900, RS, Brazil; gabrielakurowiski@hotmail.com; 2 Laboratory of Biochemical Engineering, College of Chemistry and Food Engineering, Federal University of Rio Grande, Rio Grande 96203-900, RS, Brazil; jorgealbertovc@gmail.com; 3 Center for the Development of Spirulina-Based Foods, College of Chemistry and Food Engineering, Federal University of Rio Grande, Rio Grande 96203-900, RS, Brazil; 4 Centre of Marine Sciences of the Algarve (CCMAR/CIMAR LA), Gambelas Campus, University of Algarve, 8005-139 Faro, Portugal; lbarreir@ualg.pt

**Keywords:** algae-based foods, sensorial properties, sustainable food ingredient

## Abstract

The intense green coloration of *Spirulina* biomass limits its incorporation into food products despite its high nutritional and bioactive value. This study evaluated the heterotrophic fed-batch cultivation of *Spirulina* sp. LEB 18 using glucose and sodium acetate, aiming to modulate biomass color and preserve a nutritionally relevant composition for its application as a food ingredient. After 10 days, all heterotrophic cultures exhibited attenuation of the green coloration, with total color differences greater than ΔE > 7 relative to the controls. The reduction in chlorophyll a was consistent with the observed color changes, whereas phycocyanin and carotenoid contents did not differ significantly from the autotrophic controls. Cultivation supplemented with sodium acetate fed at 0.2 g L^−1^ showed a protein composition similar to the control and was the condition that exhibited the greatest color attenuation (ΔE = 10.33) without a pronounced reduction in protein content. Cultures supplemented with glucose resulted in higher lipid and carbohydrate accumulation, reflecting the redirection of carbon toward energy storage compounds. The results demonstrate that heterotrophic cultivation of *Spirulina* sp. LEB 18 is a promising strategy to modulate biomass color and composition, expanding its applicability, particularly in food matrices where green coloration limits consumer acceptance.

## 1. Introduction

The scarcity of resources and the uncontrolled growth of the global population have raised concerns among governments, organizations, and the scientific community, particularly regarding nutrition and food security [1]. As a result, there has been a search for alternative sources that do not require soil and freshwater management, aiming at the sustainable production of foods with high nutritional value [2]. In this context, microalgae emerge as a sustainable source of biomass and food bioproducts. The use of microalgae in food production addresses five of the Sustainable Development Goals (SDGs): 2—Zero Hunger and Sustainable Agriculture; 3—Good Health and Well-Being; 9—Industry, Innovation and Infrastructure; 12—Responsible Consumption and Production; and 14—Life Below Water [3].

*Spirulina* is a filamentous cyanobacterium widely explored for food and biotechnological applications. Although taxonomically classified as a cyanobacterium, it is commonly referred to as a microalga in the food and biotechnology literature due to its historical and industrial use [4,5]. It is recognized for its potential to mitigate greenhouse gas emissions and is certified as safe for consumption by both the FDA (Food and Drug Administration) and EFSA (European Food Safety Authority) [4,6]. The biomass derived from *Spirulina* cultivation is used in the food industry as a functional ingredient because it is rich in bioactive compounds, such as antioxidant pigments, essential amino acids, and polyunsaturated fatty acids [7,8,9].

Although *Spirulina* biomass offers numerous benefits related to sustainability and health, its organoleptic characteristics present limitations for use as a food ingredient. The biomass, with its intense green coloration, frequently results in low sensory acceptance by consumers due to the impact of appearance on foods such as breads, snacks, and beverages [10,11,12,13]. Therefore, obtaining biomass with attenuated green coloration while maintaining or even enhancing its nutritional benefits may represent a strategy to improve its acceptance [14,15,16].

Modification of *Spirulina* cultivation conditions may result in changes in biomass color due to the accumulation or degradation of photosynthetic pigments. Heterotrophic cultivation, carried out in the dark and supplemented with organic carbon sources, bypasses the light-dependent phase of photosynthesis. Consequently, a reduction in chlorophyll *a* content, the main photosynthetic pigment, may occur. Changes in metabolic conditions may also affect the profile of secondary pigments, such as phycocyanin and carotenoids. In this way, modifications in the color of the produced microalgal biomass can be achieved [17,18,19]. Although several studies have investigated the effects of heterotrophic cultivation on microalgal growth and biochemical composition, few have explored the intentional modulation of biomass color as a strategy to expand the technological applicability of a *Spirulina* as a food ingredient [20,21,22,23,24,25,26,27]. Furthermore, the available literature remains limited regarding the evaluation of how different organic carbon sources simultaneously influence biomass color together with pigment composition, proximate composition, and instrumental colorimetric properties under heterotrophic fed-batch cultivation.

Thus, the development of *Spirulina* biomass with attenuated coloration represents a promising approach to reduce sensory limitations associated with its intense green color, favoring its incorporation into different food matrices without compromising the content of bioactive and nutritional compounds [9,14,16,28]. Furthermore, heterotrophic cultivation may contribute to more sustainable production processes due to the lower dependence on artificial illumination. Therefore, the objective of this study was to evaluate the heterotrophic fed-batch cultivation of *Spirulina* sp. LEB 18 using glucose and sodium acetate, aiming to modulate biomass color and preserve a nutritionally relevant composition for its application as a food ingredient.

## 2. Materials and Methods

### 2.1. Microalgae

The microalga *Spirulina* sp. LEB 18 was used for the development of this work, which is part of the strain bank of the Biochemical Engineering Laboratory of the Federal University of Rio Grande (FURG), Rio Grande, Brazil. This microalga was isolated from Lagoa da Mangueira, at latitude 33°31′08″ S and longitude 53°22′05″ W [29].

### 2.2. Cultivation Conditions

The cultures were initiated with an inoculum concentration of 0.2 g L^−1^. The experiments were conducted in 500 mL *Erlenmeyer* flasks, with a working volume of 400 mL, maintained in an orbital shaker (Innova 44, New Brunswick, Edison, NJ, USA) at 30 ± 2 °C and 100 rpm for 10 days. The cultures were carried out under heterotrophic conditions, with the shaker chamber maintained in complete darkness, in a fed-batch system using glucose (G) or sodium acetate (NaAc) as organic carbon sources, evaluated separately at concentrations of 0.2, 1.0, and 2.0 g L^−1^. Zarrouk medium [30] (Table 1) was used as the culture medium, in which the inorganic carbon source (NaHCO_3_) was replaced by the respective organic carbon source. The initial pH was adjusted to 9.5 by the addition of a sodium hydroxide solution (NaOH), the optimal pH for *Spirulina* growth. The cultures were fed every two days using 40 g L^−1^ stock solutions of glucose and sodium acetate, with the same carbon concentration corresponding to each treatment (0.2, 1.0, or 2.0 g L^−1^) added at each feeding. Four control conditions were evaluated: complete Zarrouk medium under a 12 h light/12 h dark photoperiod with white LED illumination (strips, 6500 K, 60 µmol photons m^−2^ s^−1^) (Positive Control Light, PCL); Zarrouk medium without NaHCO_3_ under a 12 h light/12 h dark photoperiod with white LED illumination (strips, 6500 K, 60 µmol photons m^−2^ s^−1^) (Negative Control Light, NCL); complete Zarrouk medium under continuous darkness (Positive Control Dark, PCD); and Zarrouk medium without NaHCO_3_ under continuous darkness (Negative Control Dark, NCD). Cultures were performed in duplicate due to the large number of experimental conditions evaluated simultaneously. This experimental design is consistent with previous heterotrophic cultivation studies of *Spirulina* [22,23,31], particularly in exploratory screening approaches conducted under controlled shaker cultivation conditions. The use of duplicate cultures allowed comparative evaluation among treatments while maintaining consistent cultivation parameters throughout the experimental period. At the end of the cultivation period, glucose concentration was determined using the reducing sugar assay with 3,5-dinitrosalicylic acid (DNS method) [32]. Because sodium acetate does not react in the DNS assay, only residual glucose was subtracted from the total carbohydrate quantification in glucose-fed cultures.

### 2.3. Growth Evaluation

Cultivations were monitored every two days from start to finish by determining pH and biomass concentration. The pH was measured using a digital pH meter (FiveGo FG2, Mettler Toledo, Greifensee, Switzerland). Cell concentration (Xcel) was determined by counting using a Neubauer chamber (*Neubauer*-Bright Line, BOECO, Hamburg, Germany). Biomass concentration (X) was determined from X_cel_, using the triple correlation between this variable, optical density (OD), and dry weight. Equations (1) and (2) were obtained using linear regression to determine X.

(1) $$\mathrm{OD}\;=4\;\times \;10^{6}\;X_{\mathrm{cel}}+0.0557\left(R^{2}=0.98\right)$$

(2) $$X\;(g\;L^{-1})=0.4628\;\mathrm{OD}\;-\;0.022\left(R^{2}=0.99\right)$$

The kinetic parameters maximum concentration (X_max_, g L^−1^) and maximum productivity (P_max_, g L^−1^ d^−1^, Equation (3)) were determined from the biomass concentration [33].

(3) $$P_{\max}=\frac{\left(X_{t}-X_{0}\right)}{\left(t-t_{0}\right)}$$

### 2.4. Pigment Screening

Scans were conducted every 5 days to assess the behavior of pigments within the cultures. To accomplish this, a graph of wavelength (400 to 700 nm) versus absorbance was plotted by measuring the optical densities of the studied samples (UV 1900, Shimadzu, Kyoto, Japan) [34]. The wavelength ranges described by Pagels et al. (2020) [35] were used as a reference for pigment identification, as follows: chlorophylls, 450–475 nm and 630–675 nm; carotenoids, 400–450 nm; and phycobiliproteins, 500–650 nm.

### 2.5. Biomass Recovery

After 10 days, the culture was centrifuged (Hitachi, Himac CR-GIII, Tokyo, Japan) at 2000× *g* for 20 min. The supernatant was discarded, and water was added to wash the pellets, thereby removing residual salt from the culture medium. The obtained biomass was then frozen at −80 °C (Eppendorf, New Brunswick Scientific Innova U535, São Paulo, Brazil) and subjected to freeze-drying (LAB CONCO, Kansas City, MO, USA) for 48 h before being stored at −20 °C until characterization.

### 2.6. Color Measurament

To identify the difference in color between the positive control culture with light (PCL), color coordinates were determined. Biomass samples obtained from the cultivations were individually distributed on circular glass supports measuring 50 mm in diameter and 10 mm in height. The L*, a*, and b* values were measured at three equidistant points using a digital colorimeter (CR-400, Konica Minolta, Tokyo, Japan) with a D65 illuminant and a 10° standard observer angle. The following parameters were determined: chroma (C*ab, Equation (4)), representing the intensity of the color; hue angle (h°ab, Equation (5)), indicating the tonal quality of the color in degrees; and total color difference (ΔE, Equation (6)), corresponding to the perceptual distance between two colors within the color space. In this context, a* represents the color component ranging from green (−) to red (+); b* represents the color component ranging from blue (−) to yellow (+); and L* denotes lightness, with values spanning from 0 (black) to 100 (white) [36].

(4) $$C^{*}ab=\sqrt{{{(a}^{*})}^{2}+{{\;(b}^{*})}^{2}}$$

(5) $$h{}^{\circ}\mathrm{ab}=\arctan\left(\frac{b^{*}}{a^{*}}\right)$$

(6) $$∆E=\sqrt{{\left({∆L}^{*}\right)}^{2}+{\left({∆a}^{*}\right)}^{2}+{\left({∆b}^{*}\right)}^{2}}$$

### 2.7. Biomass Composition

The lyophilized biomass was used for lipid determination following the method described by Marsh and Weinstein [37]. For protein [38] and carbohydrate [39] determinations, extracts were obtained from the biomass. The extracts were prepared by using 5 mg of biomass and 10 mL of distilled water, which were subjected to an ultrasonic probe (CPX-130, COLE PARMER, Vernon Hills, IL, USA) for 10 min, in cycles of 59 s (On/Off).

### 2.8. Pigment Quantification

The same methodology for obtaining the extract described in Section 2.7 was employed to disrupt the cell wall of *Spirulina* sp. LEB 18. 10 mL of the extract were mixed with 10 mL of a 6 g L^−1^ calcium chloride solution, and the mixture was subjected to magnetic stirring for 1 h. Subsequently, the samples were centrifuged at 10,000× *g* for 5 min (Hitachi, Himac CR-GIII, Tokyo, Japan), resulting in two fractions that will be used for the determination of phycocyanin, chlorophyll *a*, and carotenoids [40].

#### 2.8.1. Phycocyanin Content

The supernatant obtained from the centrifugation described in Section 2.8 was used to determine the phycocyanin content by measuring the optical density (UV 1900, Shimadzu, Kyoto, Japan) at wavelengths 615 and 652 nm. Equation (7) shows the correlation used to determine the phycocyanin content (mg mL^−1^) proposed by Bennett and Bogorad [41], where Abs_615_ and Abs_652_ correspond to the absorbances measured at the respective subscript wavelengths. The results were expressed in mg g^−1^ of biomass, considering the mass of biomass used for extraction and the corresponding volumes involved in the analysis.

(7) $$\mathrm{Phycocyanin}\;(\mathrm{mg}\;{\mathrm{mL}}^{-1})\;=\;\frac{{\mathrm{Abs}}_{615}-0.474\left({\mathrm{Abs}}_{652}\right)}{5.34}$$

#### 2.8.2. Chlorophyll and Carotenoid Content

Using the pellets obtained from the centrifugation described in Section 2.8, 10 mL of 80% (*v*/*v*) acetone was added, followed by vortex homogenization. The mixture was centrifuged (10,000× *g* 5 min), and the supernatant was collected. This procedure was repeated until the pellets became white or grayish, indicating complete pigment extraction. The number of extraction cycles was recorded for subsequent consideration in the calculation of chlorophyll and carotenoid contents. At the end of procedure, the collected supernatants (concentrated extract) were pooled, and the optical density of the concentrated extract was determined at wavelengths of 470, 646.6, and 663.6 nm [40]. The chlorophyll *a* and carotenoid content (mg mL^−1^) were determined using the correlations established by Lichtethaler [42], described in Equations (8) and (9), respectively, whereas in the equations, Abs_470_, Abs_646_._6_, and Abs_663_._6_ correspond to the absorbances obtained at the respective subscript wavelengths. The results were expressed in mg g^−1^ of biomass, considering the mass of biomass used for extraction and the corresponding volumes involved in the analysis.

(8) $$\mathrm{Chlorophyll}\;a\;(\mathrm{mg}\;{\mathrm{mL}}^{-1})=\frac{12.25\left({\mathrm{Abs}}_{663.6}\right)-2.55\left({\mathrm{Abs}}_{646.6}\right)}{1000}$$

(9) $$\mathrm{Carotenoids}\;(\mathrm{mg}\;{\mathrm{mL}}^{-1})=\frac{1000\left({\mathrm{Abs}}_{470}\right)-1.82\left(\mathrm{Chlorophyll}\;a\right)}{1.98\;\times \;10^{5}}$$

### 2.9. Statistical Analysis

The cultures were conducted in biological duplicates, with each duplicate representing an independent cultivation under identical experimental conditions. All analyses, biochemical and colorimetric, were performed in technical triplicates, resulting in six analytical replicates per condition (*n* = 6). Results are expressed as mean ± standard deviation (SD). Statistical significance was assessed by one-way analysis of variance (ANOVA), followed by the Tukey test, with a 95% confidence level (*p* < 0.05). Statistical analyses were performed using the Statistica 7 software (StatSoft Inc., Tulsa, OK, USA).

## 3. Results

### 3.1. Microalgal Growth and Biomass Production

The growth parameters of *Spirulina* sp. LEB 18 are presented in Table 2. The autotrophic controls showed higher biomass concentrations. The positive control under light conditions (PCL) exhibited growth associated with the presence of light and inorganic carbon (NaHCO_3_). In the negative control under light conditions (NCL), cellular development was observed even in the absence of carbon in the medium. In the heterotrophic controls under absence of light, negative (NCD) and positive (PCD), the cultures remained viable for up to 4 days.

**Figure 1:**
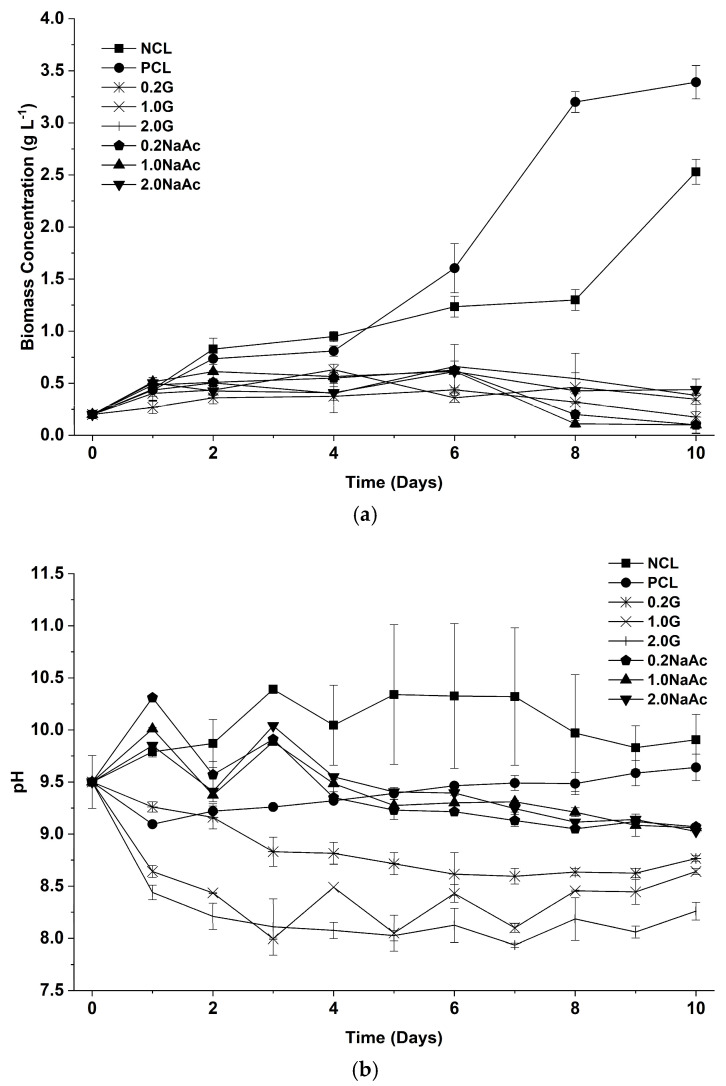
Growth curves (**a**) and pH profile (**b**) of *Spirulina* sp. LEB 18 under control and heterotrophic conditions over 10 days of cultivation. (NCL) Negative Control Light, (PCL) Positive Control Light, (0.2 G) 0.2 g L^−1^ Glucose, (1.0 G) 1.0 g L^−1^ Glucose, (2.0 G) 2.0 g L^−1^ Glucose, (0.2 NaAc) 0.2 g L^−1^ Sodium Acetate, (1.0 NaAc) 1.0 g L^−1^ Sodium Acetate, and (2.0 NaAc) 2.0 g L^−1^ Sodium Acetate.

The highest P_max_ were obtained in cultures supplemented with sodium acetate (NaAc) and glucose (G) at 2.0 g L^−1^ (2.0 G), with X_max_ values that did not differ significantly between these assays (*p* < 0.05). Under all heterotrophic conditions, biomass concentrations did not exceed 1 g L^−1^. The growth curves of the NCL and PCL controls presented well-defined phases (Figure 1a). The pH varied according to the substrate used (Figure 1b). In glucose cultures, values ranged from 7.9 to 9.5, whereas in NaAc cultures they ranged from 9.0 to 10.6. On days without supplementation, an increasing trend in pH was observed.

### 3.2. Pigmentation Dynamics During Cultivation

**Figure 2:**
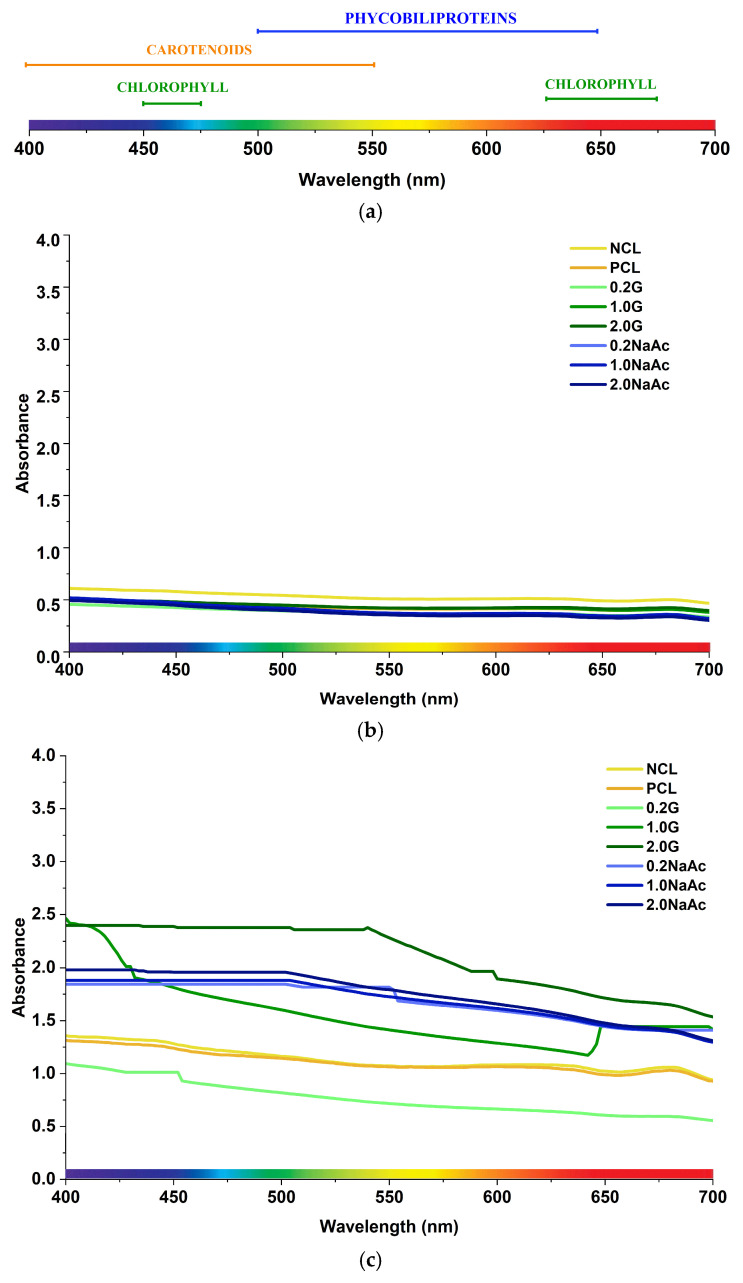

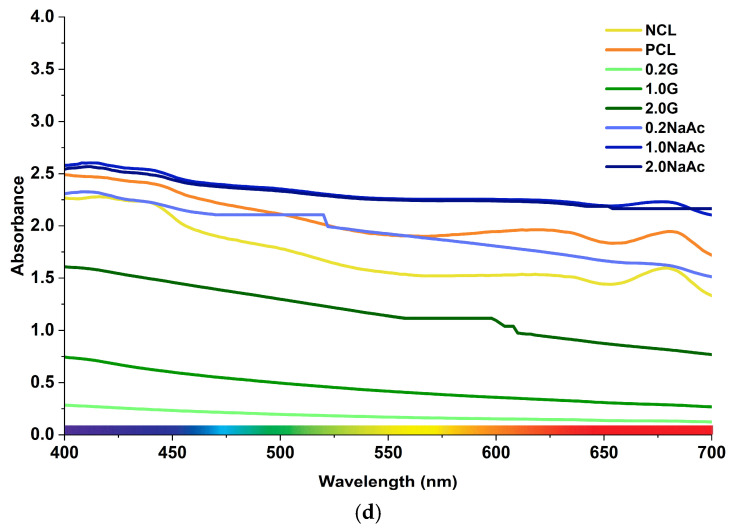
Screening of pigments over 10 days of cultivation of *Spirulina* sp. LEB 18: (**a**) Reference Bands for Pagels et al. [35], (**b**) Time 0, (**c**) Time 5, (**d**) Time 10.

The results of pigment analyses throughout the experimental period are presented in Figure 2. It was observed that G and NaAc distinctly influenced the absorbance, which may be attributed to the levels of chlorophyll, phycocyanin, and carotenoids. Light exposure may have promoted pigment accumulation in both the NCL and the PCL, whereas heterotrophic conditions resulted in variations in pigment biosynthesis throughout cultivation. Glucose supplementation, particularly at the concentration of 2.0 g L^−1^ (2.0 G), favored earlier pigment synthesis, with an increase observed on the fifth day of cultivation (Figure 2b). In contrast, treatments supplemented with sodium acetate presented a gradual increase in pigments throughout the 10 experimental days (Figure 2c). In *Spirulina* sp. LEB 18 cultures, an increase in pigment-associated absorbance was observed on the fifth day under glucose supplementation, followed by a subsequent decline. On the other hand, cultures supplemented with sodium acetate exhibited a progressive increase in pigment levels throughout the cultivation period.

### 3.3. Nutritional Profile of Biomass

The proximal composition of the biomass obtained after 10 days of cultivation is presented in Table 3. Regarding macromolecules, the highest protein contents were observed under PCL (38.68%) and heterotrophic 0.2 NaAc (32.18%) conditions, followed by 2.0 NaAc (26.87%) and 1.0 G (23.38%). For carbohydrates, the highest concentrations were recorded in the PCL (17.46%) and NCL (17.36%) controls. Among the heterotrophic conditions, the highest value was obtained in 2.0 G (14.47%). In general, conditions supplemented with sodium acetate showed lower carbohydrate contents, except for 0.2 NaAc. Regarding lipids, the values did not differ statistically (*p* > 0.05) from the controls (PCL and NCL), except for the 0.2 G and 2.0 NaAc conditions, which presented higher levels.

### 3.4. Pigment Profile of Spirulina Biomass

**Figure 3:**
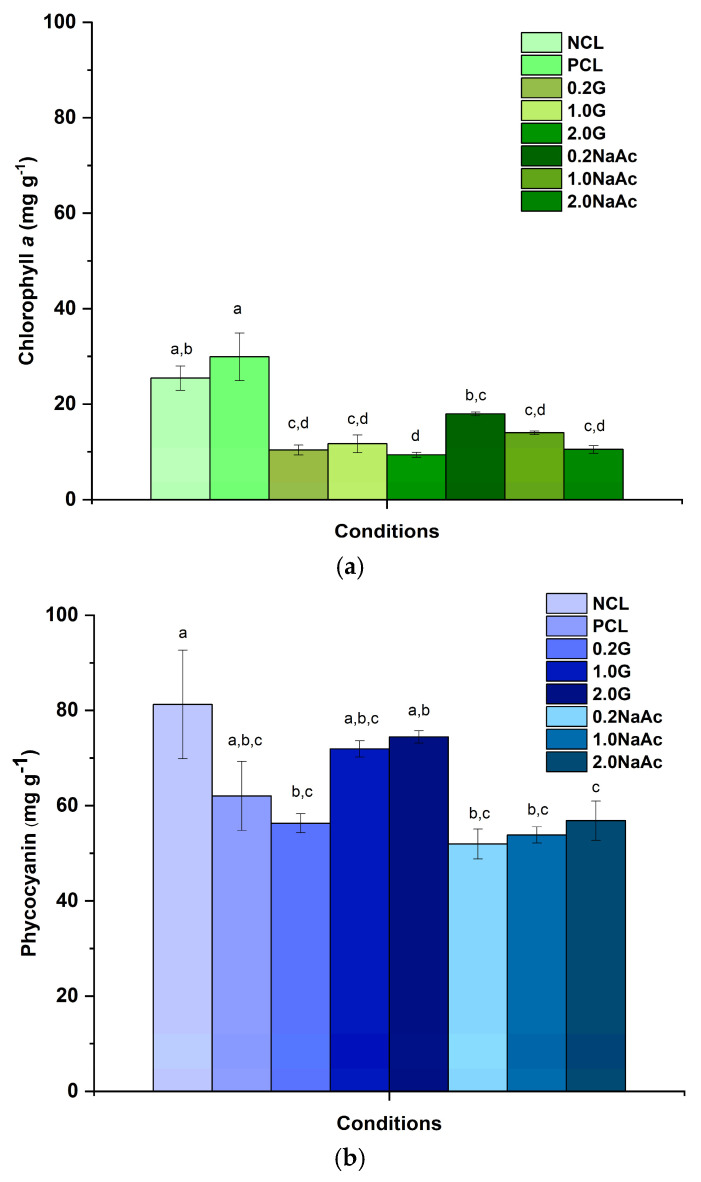

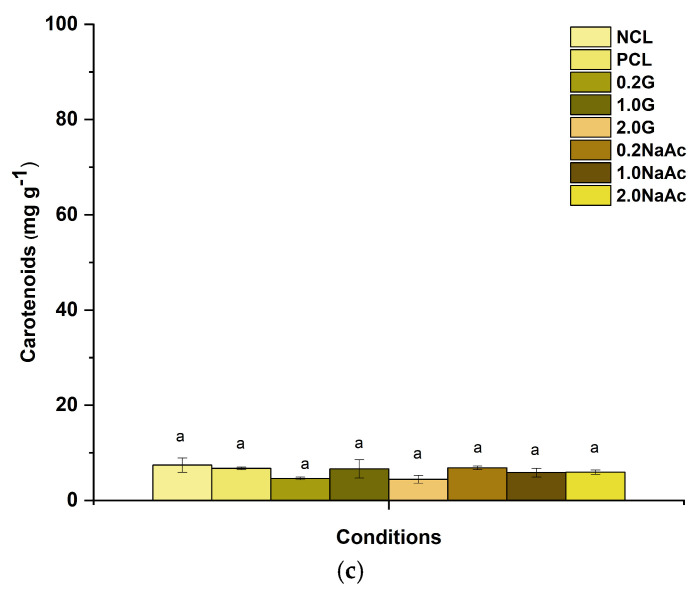
Pigment concentrations: (**a**) chlorophyll a, (**b**) phycocyanin, and (**c**) carotenoids in *Spirulina* sp. LEB 18 biomass cultivated for 10 days. Letras diferentes indicam diferenças significativas entre os tratamentos, enquanto letras iguais indicam ausência de diferenças significativas (*p* < 0.05).

The results of pigment quantification in the final biomass of *Spirulina* sp. LEB 18 after 10 days of cultivation are presented in Figure 3. Chlorophyll *a* content (Figure 3a) in cultures supplemented with G and NaAc differed significantly (*p* < 0.05) in relation to the PCL control (29.1 ± 5.0 mg g^−1^)m, with values ranging from 9.4 ± 0.5 mg g^−1^ (2.0 G) to 18.0 ± 0.4 mg g^−1^ (0.2 NaAc). In contrast, the NCL control presented chlorophyll *a* levels similar to those of PCL (25.5 ± 2.5 mg g^−1^). Phycocyanin concentration (Figure 3b) did not show a statistically significant difference (*p* > 0.05) in relation to PCL (62.0 ± 7.2 mg g^−1^) for all evaluated concentrations of G and NaAc, which ranged from 52.0 ± 3.1 mg g^−1^ (0.2 NaAc) to 74.4 ± 1.2 mg g^−1^ (2.0 G); the highest phycocyanin content among all conditions was observed in NCL (81.3 ± 11.4 mg g^−1^). Carotenoid concentration (Figure 3c) remained statistically similar (*p* > 0.05) under all studied conditions, with values ranging from 4.45 ± 0.81 mg g^−1^ (2.0 G) to 7.44 ± 1.15 mg g^−1^ (NCL).

### 3.5. Color Measurements and Biomass Visual Apperance

Table 4 presents the color space parameters of *Spirulina* sp. LEB 18 biomass after 10 days of cultivation. For lightness (L*), the NCL (36.71) and PCL (37.95) assays resulted in lower values, indicating darker coloration. The 2.0 NaAc condition differed significantly from the controls, presenting higher L* values. The a* parameter, related to the red–green axis, presented negative values under all conditions, indicating the predominance of green tones. In cultures supplemented with glucose, a tendency toward lighter green shades was observed, especially at the concentration of 1 g L^−1^. In cultures supplemented with sodium acetate, increasing concentration resulted in cells with lighter green coloration. For the b* parameter, corresponding to the blue–yellow axis, positive values were observed under all conditions. Cultures supplemented with NaAc at concentrations of 0.2 and 1 g L^−1^ presented higher b* values compared to the control, indicating a more yellowish coloration. In contrast, cultures supplemented with glucose exhibited reduced b* values, particularly at higher concentrations.

**Figure 4:**
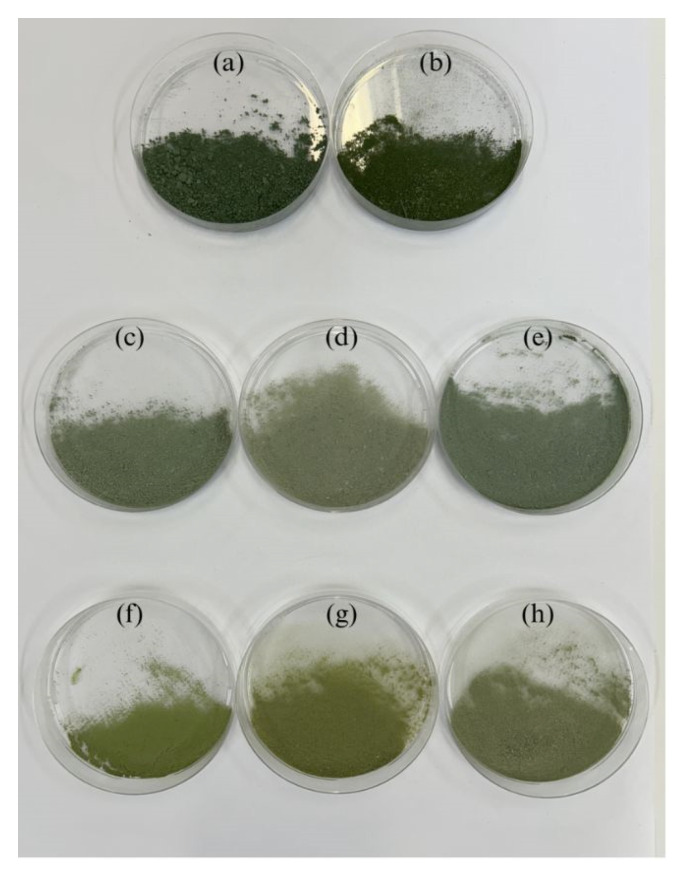
*Spirulina* sp. LEB 18 biomass obtained from controls and heterotrophic cultures with glucose and sodium acetate supplementation at different concentrations: (**a**) Negative Control Light, (**b**) Positive Control Light, (**c**) 0.2 g L^−1^ Glucose, (**d**) 1.0 g L^−1^ Glucose, (**e**) 2.0 g L^−1^ Glucose, (**f**) 0.2 g L^−1^ Sodium Acetate, (**g**) 1.0 g L^−1^ Sodium Acetate and (**h**) 2.0 g L^−1^ Sodium Acetate.

Table 5 presents the chroma (C*ab), hue angle (h°ab), and total color difference (ΔE) values. The highest C*ab values were observed in cultures supplemented with NaAc, particularly at concentrations of 0.2 and 1 g L^−1^. In cultures supplemented with glucose, the C*ab values were lower. The h°ab values indicated variations among the conditions, except for the 1.0 G and 2.0 NaAc assays. In NaAc-supplemented cultures, especially at concentrations of 1 and 2 g L^−1^, values associated with cooler tones were observed. In glucose-supplemented cultures, the concentration of 1 g L^−1^ presented a greater shift toward green compared to the other concentrations. The total color difference (ΔE), calculated in relation to the PCL control, presented the lowest value for NCL. The NaAc and glucose conditions exhibited perceptible differences, with the highest value observed for 2.0 NaAc. Figure 4 presents the visual appearance of the biomass, highlighting the color variations among the studied conditions.

## 4. Discussion

### 4.1. Impact of Carbon Sources on Biomass Production and Growth Behavior

The results highlight the influence of cultivation conditions and carbon sources on the growth of *Spirulina* sp. LEB 18. The higher biomass production in the autotrophic controls confirms the essential role of light and inorganic carbon in photosynthetic metabolism, in which NaHCO_3_ is converted into CO_2_ and assimilated during the light phase. In contrast, the growth limitation observed in heterotrophic controls under absence of light (NCD and PCD) reinforces the dependence of the *Spirulina* on adequate energy sources, whether luminous or organic. In the case of NCL, the observed growth may be associated with the utilization of intracellular carbon reserves [43,44]. Although heterotrophic productivities were lower than autotrophic ones, the obtained values were sufficient to characterize the biomass in terms of composition and color, which are central parameters for its application as a food ingredient.

The higher productivity obtained in heterotrophic cultivations supplemented with NaAc and 2.0 G indicate that the efficiency of carbon source assimilation depends on its physicochemical properties and the metabolic capacity of the strain. In studies involving sodium acetate and cyanobacteria, which may be associated with *Spirulina*, dissociation in liquid medium may have provided greater availability for cellular uptake, whereas glucose, which does not dissociate, may have depended more strongly on diffusion and mixing conditions of the medium [45,46,47]. This behavior is consistent with observations that different carbon sources result in variations in maximum concentrations and yields in heterotrophic cultivations [17].

The observed pH variations directly reflect the metabolism of the substrates used. Glucose may lead to the formation of organic acids, promoting acidification of the medium, whereas sodium acetate acts as a buffering agent, contributing to the maintenance of alkaline values. Furthermore, the formation of carbonic acid (H_2_CO_3_) from the metabolism of these carbon sources may also influence system pH [17,19,48]. Even under conditions that are not strictly anaerobic, the production and consumption of organic acids, such as succinic acid, may occur, contributing to the observed fluctuations [18,22,44,49,50,51,52].

The alkalizing character of NaAc, associated with the formation of carbonates and bicarbonates, explains the increase in pH on days without supplementation, possibly due to the presence of residual acetate or metabolic products in the medium [48]. Although pH values remained close to the optimal range for *Spirulina* (9.5–9.8), daily fluctuations may have negatively impacted growth [5]. Feeding every two days contributed to attenuating pH oscillations, reducing the potential stress on the cells, although it was not sufficient to eliminate them completely.

Thus, the limitation of biomass concentrations under heterotrophic conditions (<1 g L^−1^) may be related to stress caused by pH, absence of light, and possibly by the consumption of all available nitrogen in the culture medium. Nitrogen is an essential component of enzymes responsible for metabolic processes [53]. Strategies such as the addition of solutions for pH correction, gradual reduction in light intensity, and fed-batch supplementation with sodium nitrate could minimize cellular stress and improve biomass conversion [54,55]. Consequently, productivity would be increased, improving the feasibility of *Spirulina* sp. LEB 18 biomass production. The biomass characterized in terms of pigmentation dynamics and proximal composition, which are determining aspects for its application as a food ingredient, will be discussed in the following sections.

### 4.2. Role of Heterotrophic Cultivation in Modulating Spirulina Pigmentation

The differences observed in chlorophyll *a* and phycocyanin dynamics indicate that G and NaAc distinctly modulate the pigment metabolism of *Spirulina* sp. LEB 18 in response to the intracellular C/N balance [53,56]. Glucose assimilation under heterotrophic conditions occurs through the pentose phosphate (PP) and Embden–Meyerhof (EM) pathways [27,57]. Sodium acetate is metabolized by cyanobacteria through conversion into acetyl-CoA via acetyl-CoA synthetase [47]. The earlier onset of pigment synthesis under glucose supplementation may be related to the increase in cellular growth rate, which elevates the nitrogen demand for protein synthesis. This increase in demand may lead to faster consumption of the available nitrogen, resulting in nutritional limitation and subsequent reduction in pigment levels. In contrast, sodium acetate, when metabolized through acetyl-CoA formation, tends to exert lower immediate pressure on nitrogen assimilation, allowing a more balanced distribution of metabolic fluxes and a more gradual increase in pigmentation [14,18,19,53].

Furthermore, the absence of light constitutes a relevant factor in pigment modulation. Under prolonged heterotrophic conditions, a reduction in the content of photosynthetic pigments is frequently observed, reflecting the lower functional demand for these compounds [58,59,60]. In this context, the decline observed after the initial peak under glucose supplementation may be associated with both nitrogen limitation and the reduced requirement for maintaining an active photosynthetic apparatus. Overall, the results suggest that glucose promotes faster and more intense metabolic responses, whereas sodium acetate favors a more stable and progressive behavior in pigment biosynthesis, highlighting distinct metabolic adaptation strategies to the available carbon sources. Thus, the temporal modulation of photosynthetic pigments during heterotrophic cultivation indicates that it is possible to obtain biomasses with different pigmentation profiles and, consequently, different colorations through adjustment of the carbon source and concentration, representing a viable strategy to tailor the visual characteristics of the biomass to the requirements of different food matrices.

### 4.3. Effects of Heterotrophic Cultivation on Biomass Nutritional Quality

The results indicate that the carbon source exerts a direct influence on the biochemical composition of *Spirulina* sp. LEB 18 biomass. The protein content obtained in the present study for PCL (38.68%) was lower than the values of 50–70% commonly reported for commercial *Spirulina* cultivated under photoautotrophic conditions [15,61]. This difference should be interpreted with caution, as the values reported in the literature generally refer to crude protein determined by nitrogen-based methods, such as Kjeldahl or Dumas, which tend to generate overestimated values due to the application of a fixed conversion factor (6.25) [62], although lower conversion factors have been specifically recommended for microalgal biomass [63,64]. In the present study, protein content was quantified using the Lowry method [38], which is based on the reaction with aromatic amino acid residues and peptide bonds rather than on the indirect quantification of total nitrogen, which may explain part of the discrepancy observed. Furthermore, the photoautotrophic cultivations were performed in *Erlenmeyer* flasks under orbital shaking and low light intensity, conditions that may limit mass transfer and light homogeneity compared with optimized cultivation systems [4,65].

The higher protein content observed under sodium acetate conditions, especially in 0.2 NaAc, suggests that this carbon source favors amino acid biosynthesis. This behavior may be explained by the acetate metabolic pathway, in which acetate is directly converted into acetyl-CoA and integrated into the tricarboxylic acid (TCA) cycle, generating intermediates such as α-ketoglutarate and oxaloacetate, which are precursors for amino acid synthesis. In contrast, glucose requires additional conversion steps through glycolysis to pyruvate before entering the TCA cycle [14,18,19,38].

The higher carbohydrate contents in the illuminated controls (PCL and NCL) reflect the predominance of photosynthetic pathways in carbon assimilation, favoring polysaccharide formation. Among the heterotrophic conditions, the prominence of 2.0 G indicates that glucose, being directly associated with the glycolytic pathway, provides immediate precursors for the synthesis of structural and storage carbohydrates, such as glycogen. In contrast, the lower performance of acetate in carbohydrate synthesis suggests reduced metabolic flux toward this pathway under the evaluated conditions [14,19,48,66].

Regarding lipids, the absence of significant differences under most conditions indicates that the lipid pathway was not strongly favored. However, the increase observed in 0.2 G suggests that low glucose concentrations may stimulate energy storage in the form of lipids. Similarly, the increase observed in 2.0 NaAc may be related to the higher availability of acetyl-CoA, a direct precursor of fatty acid synthesis, thus favoring lipogenesis [17,18,19]. The absence of lipid increase under the other conditions may indicate metabolic limitations or rapid intracellular utilization of the produced lipids. Furthermore, the possible limitation of nutrients such as nitrogen and magnesium, associated with heterotrophic cultivation, may have influenced lipid conversion [67].

For *Spirulina* sp. LEB 18 biomass to be incorporated into foods, it must present a nutritional composition competitive with commercially available biomass. According to AlFadhly et al. [68], the commercial composition ranges from 55–72% proteins, 13–25% carbohydrates, and 4–8% lipids (dry weight). Mohammed et al. [69] studied *Spirulina maxima* biomass incorporated into a milk beverage characterized by containing 59.08 ± 1.01% proteins, 3.12 ± 0.48% lipids, and 19.11 ± 1.24% carbohydrates. Bordulis et al. [70] characterized commercial *Spirulina maxima* for the development of functional shakes for elderly individuals, in which the biomass contained 60.3% proteins. Paternina et al. [71] used *Spirulina* sp. LEB 18 as an ingredient for the development of gummy candies, in which the biomass contained 58.53 ± 0.73% proteins, 15.51 ± 0.32% lipids, and 11.13 ± 0.03% carbohydrates. Rasia et al. [72] developed a chocolate milk enriched with nanocapsules of *Spirulina* sp. LEB 18, in which the biomass used as an ingredient contained 41.37 ± 0.45% proteins, 22.80 ± 1.49% lipids, and 22.30 ± 1.09% carbohydrates.

The results described above demonstrate that the composition of *Spirulina* sp. LEB 18 resulted in lower macromolecule contents when compared with commercial biomasses used as food ingredients obtained through autotrophic cultivation. Cruz et al. [31], who performed heterotrophic fed-batch cultivation of *Spirulina* sp. LEB 18 supplemented with 0.5 g L^−1^ glucose, obtained 39.8 ± 1.4% proteins, 12.0 ± 0.8% lipids, and 6.4 ± 0.6% carbohydrates. Similar to Cruz et al. [31], the present study resulted in reduced macromolecule production due to the combination of absence of light and organic carbon supplementation. Thus, under heterotrophic conditions, the results likely reflect the interaction of multiple limiting factors, including pH fluctuations, lower energy availability from photosynthesis, and potential nutritional imbalances inherent to the cultivation design [73]. Although previous studies with *Spirulina* sp. LEB 18 have reported the accumulation of storage biopolymers, such as polyhydroxybutyrate (PHB), under heterotrophic conditions [74], the present study did not evaluate these compounds. Future studies incorporating PHB quantification would help clarify whether unaccounted biomass fractions correspond to such storage products and would provide a more complete metabolic understanding of carbon partitioning under heterotrophic cultivation.

### 4.4. Heterotrophic Modulation of Pigment Composition

The reduction in chlorophyll *a* content under heterotrophic conditions is associated with the absence of light, which compromises the maintenance of the photosynthetic machinery. The interruption of pigment synthesis, combined with the possible degradation of photosystems over time, explains the lower values observed under these conditions [58,59,60]. In contrast, the maintenance of chlorophyll *a* levels in the NCL control, similar to PCL, reinforces the role of light as a determining factor for the stability of this pigment [17]. The reduction in chlorophyll *a* content is the primary mechanism responsible for the attenuation of the green coloration of *Spirulina* sp. LEB 18 biomass, which will be discussed in Section 4.5.

The stability of phycocyanin among the different conditions, including heterotrophic cultivations, indicates that the production of this accessory pigment was not significantly affected by the absence of light or by the carbon source used. Although phycocyanin acts in photon capture at wavelengths not efficiently absorbed by chlorophyll *a*, its maintenance suggests that, under the evaluated conditions, there was neither sufficient stimulus for its overexpression nor pronounced degradation. In prolonged heterotrophic cultivations, reduction in the antenna complex may occur, leading to the inactivation of this pigment; however, this effect was not significantly observed in the present study [58,60,75]. The pigment phycocyanin is commercially applied due to its bioactive characteristics, such as antioxidant and antibacterial activities, in ice cream [76], wine, isotonic drinks, and tonics [77] and gelatin-based films [78]. The preservation of phycocyanin content ensures that the bioactivity associated with this pigment is maintained.

The chlorophyll a and phycocyanin contents obtained in the present study are consistent with the trend reported in previous studies involving the heterotrophic cultivation of *Spirulina* species. Marquez et al. [22] evaluated the heterotrophic cultivation of *Spirulina platensis* NIES-39 supplemented with 2 g L^−1^ glucose in batch culture and reported a marked reduction in both chlorophyll *a* content (5.61 mg g^−1^ under heterotrophic conditions vs. 10.84 mg g^−1^ under autotrophic conditions) and phycocyanin content (57.9 mg g^−1^ vs. 122 mg g^−1^, respectively), corroborating the pattern observed under the G and NaAc conditions in the present study relative to the PCL and NCL controls. Similar results were reported by Marquez et al. [23], who investigated the mixed (heterotrophic/autotrophic) cultivation of *Spirulina platensis* NIES-39 in SOT medium supplemented with 2 g L^−1^ glucose. During the heterotrophic phase, phycocyanin and chlorophyll *a* contents reached 55.2 mg g^−1^ and 5.33 mg g^−1^, respectively, values substantially lower than those obtained during the autotrophic phase under 82 µmol photons m^−2^ s^−1^ (157.2 mg g^−1^ phycocyanin and 29.9 mg g^−1^ chlorophyll a). This behavior is consistent with that observed in the present study, in which the absence of light under heterotrophic conditions resulted in lower chlorophyll *a* contents than those measured in the PCL and NCL controls. However, unlike chlorophyll *a*, the phycocyanin content in the present study remained relatively stable between autotrophic and heterotrophic conditions, with values even exceeding those of the PCL control at some glucose and sodium acetate concentrations (e.g., 74.45 mg g^−1^ in 2.0 G), although remaining lower than that observed for the NCL control (81.26 mg g^−1^), which exhibited the highest phycocyanin content among all conditions evaluated. This pattern differs from the pronounced reduction reported by Marquez et al. [22,23] and suggests that the response of phycobiliprotein synthesis to heterotrophic cultivation may vary according to the strain, carbon source, and specific cultivation conditions. Chen et al. [79] also demonstrated that supplementation with glucose and acetate can promote phycocyanin production in *Spirulina platensis* UTEX 1926, reinforcing the feasibility of using these organic substrates as a strategy to maintain the bioactivity associated with this pigment, even under low-light or light-free conditions.

The similarity in carotenoid contents between autotrophic and heterotrophic conditions may be attributed to the absence of high light stress. Since these pigments perform a photoprotective function, their synthesis tends to be stimulated under conditions of high light intensity. The application of intensities lower than 100 µmol photons m^−2^ s^−1^ under the control conditions was likely insufficient to induce an increase in carotenoid production, resulting in comparable levels among the different treatments [20,80].

In view of the above, *Spirulina* sp. LEB 18 biomass obtained through heterotrophic fed-batch cultivation may be economically relevant by eliminating the demand for artificial illumination, thereby reducing electricity costs associated with production. As will be discussed below (Section 4.5), this strategy may be even more favorable when considering the sensory perspective, since the attenuation of the characteristic coloration of *Spirulina* under heterotrophic conditions tends to favor consumer acceptance of the product, potentially compensating for the reduction in protein content observed under this cultivation mode.

### 4.5. Heterotrophic Modulation of Biomass Color Characteristics

The variations in color parameters reflect changes in the pigment composition of the biomass in response to cultivation conditions and carbon sources. The lower L* values observed in the autotrophic controls indicate greater color intensity, associated with the higher presence of photosynthetic pigments, especially chlorophyll a, whose synthesis is favored by the presence of light. In contrast, the increase in L* under the 2.0 NaAc condition suggests a reduction in pigment density, resulting in lighter biomass [81]. The predominance of negative a* values confirm the maintenance of green tones under all conditions, a characteristic feature of *Spirulina*. However, the shift toward lighter green tones under heterotrophic conditions, both with glucose and acetate, is consistent with the observed reduction in chlorophyll *a* concentration, indicating lower intensity of photosynthetic pigmentation [82]. The b* results indicate that sodium acetate favored more yellowish tones, especially at intermediate concentrations, whereas glucose reduced this characteristic. This behavior does not directly follow the proportion between phycocyanin and carotenoids, suggesting that the final coloration of the biomass does not depend exclusively on pigment concentration, but also on physical and structural factors such as light scattering, cellular organization, and optical interactions among the components [83,84].

The higher color saturation (C*ab) observed in NaAc cultures indicates greater color vividness, possibly associated with a more homogeneous distribution of pigments or differences in cellular composition. In contrast, the lower values observed in glucose cultures indicate a more opaque or grayish coloration, reflecting changes in the balance between pigments and other cellular constituents [85]. Variations in hue angle (h°ab) reinforce the effect of the carbon source on color modulation. The shift toward cooler tones (green–bluish) in NaAc cultures suggests changes in the relative pigment profile or their expression, whereas the effect observed with glucose was concentration-dependent, being more evident at 1 g L^−1^ [82]. ΔE values can be associated with the human perception scale, in which values higher than 6 indicate an obvious color difference [86]. All *Spirulina* sp. LEB 18 biomass obtained from heterotrophic cultivation presented humanly distinguishable color differences when compared to the control, which is explained by the reduction in chlorophyll *a* content discussed previously. The biomass from the 2.0 NaAc cultivation presented the greatest color difference, probably due to the greater contribution of b (yellowish coloration). The color variation observed in all biomasses indicates the capacity of carbon sources to modulate the visual appearance of the biomass, while the absence of light during cultivation more intensely influenced the expression and proportion of cellular pigments.

Color directly interferes with consumer perception of foods, since it is the first characteristic visually observed [81]. The visual impact of color and appearance may influence other sensory attributes of foods. Visual observation generates expectations, making color a decisive factor before the tasting experience. Thus, color is one of the dominant characteristics determining food consumption [87]. Hafsa et al. [11] developed bread enriched with *Spirulina* containing 1 and 3% biomass and obtained higher sensory acceptance (~70%) for the formulation with the lower *Spirulina* concentration. The color attribute presented the greatest difference compared to the control and was less appreciated. Zouari et al. [88] produced pasta supplemented with *Spirulina platensis*, containing 1, 2, and 3 g of biomass per 100 g of semolina. Increasing *Spirulina* concentration contributed to pasta darkening (L* = 78.08 → 52.97) and intense green coloration (a* = −11.12 ± 0.3) at the 3% concentration. In terms of overall acceptance score, considering a maximum score of 5.0, the pasta formulations containing *Spirulina* biomass presented an average score of 3.2.

Lucas et al. [12] developed extruded snacks containing 2.6% *Spirulina* sp. LEB 18 biomass. The incorporation of *Spirulina* as an ingredient significantly darkened the snacks (L* = 34.13) compared to the control (L* = 63.4), resulting in a color difference of 30.5. In this study, color was the only attribute with a significant difference compared to the control, with scores of 5.5 (*Spirulina*) and 7.6 (control) on a 9-point hedonic scale. However, purchase intention was not negatively affected. Paternina et al. [71] produced gelatin gummies containing *Spirulina* sp. LEB 18 at concentrations of 1%, 3%, and 5% biomass. Increasing biomass concentration in the formulation resulted in a gradual increase in green color intensity (a*: 1% = −0.03; 3% = −0.54; 5% = −1.39) and ΔE (1% = 30.32; 3% = 65.28; 5% = 66.60). Nevertheless, the color attribute did not interfere with sensory acceptance scores among the formulations. Lafarga et al. [89] developed broccoli soups enriched with *Spirulina* sp. at concentrations of 0.5, 1, 1.5, and 2%. It was observed that increasing *Spirulina* biomass concentration resulted in darker soup colors (L* < 39), which reduced acceptance. Application in a green matrix favored the incorporation of *Spirulina*. However, increasing *Spirulina* concentration in the soups resulted in reduced acceptance percentages (80% → 56.7%). In this sense, the color parameters obtained under the different cultivation conditions may be directly related to the behavior observed in studies involving the incorporation of *Spirulina* into foods, in which biomass coloration played a determining role in the sensory acceptance of the developed products.

Studies such as those by Lucas et al. [12], Lafarga et al. [89], Batista et al. [10], Paternina et al. [71], Zouari et al. [88], and Hafsa et al. [11] consistently demonstrated that lower L* values and more negative a* values, characteristic of intensely green biomass, tend to reduce the visual and sensory acceptance of products, especially at higher concentrations. In contrast, biomasses with more attenuated tones, such as those obtained in sodium acetate cultivations, present color parameters closer to those observed in formulations with higher acceptability reported in the literature, with acceptance indices above 70%. Therefore, modulation of biomass coloration through heterotrophic cultivation strategies emerges not only as a technological resource, but also as a consumer-oriented approach, contributing to overcoming one of the main barriers to the incorporation of *Spirulina* as a functional ingredient in the food industry.

From an industrial perspective, these findings demonstrate that heterotrophic cultivation, through appropriate carbon source selection, can be used not only to enhance biomass production but also as a process strategy to tailor the visual characteristics and biochemical composition of Spirulina biomass for specific food applications. By selecting appropriate carbon sources, it is possible to obtain biomass with distinct color profiles while maintaining a nutritionally relevant composition, thereby expanding its potential use in food products in which the characteristic intense green coloration of Spirulina may limit consumer acceptance. In addition, the reduced reliance on artificial illumination under heterotrophic cultivation has the potential to increase operational flexibility and reduce energy demand during cultivation. Overall, these results highlight the potential of heterotrophic cultivation as a versatile and sustainable platform for producing Spirulina biomass with tailored technological, nutritional, and visual attributes, broadening its applicability as a value-added ingredient for the food industry.

## 5. Conclusions

Heterotrophic fed-batch cultivation successfully modulated the color and biochemical profile of *Spirulina* sp. LEB 18 biomass through the use of glucose and sodium acetate as organic carbon sources. Light deprivation promoted chlorophyll *a* reduction, leading to attenuation of the characteristic green coloration, while phycocyanin and carotenoid contents remained comparatively stable. Among the evaluated conditions, sodium acetate supplementation, particularly at 2.0 g L^−1^, produced the greatest perceptible color difference, whereas 0.2 g L^−1^ sodium acetate preserved protein content closer to the autotrophic control. Glucose supplementation favored carbohydrate and lipid accumulation, demonstrating that carbon source selection directly influences biomass composition and visual characteristics. Although biomass productivity under heterotrophic conditions remained lower than autotrophic cultivation, the elimination of artificial illumination and the production of visually attenuated biomass represent promising advantages for food applications. Overall, the results demonstrate that heterotrophic cultivation is a feasible strategy to tailor *Spirulina* sp. LEB 18 biomass for incorporation into food matrices in which intense green coloration negatively affects consumer acceptance. Future studies should focus on process optimization and sensory validation of food products formulated with the color-modulated biomass.

## Figures and Tables

**Table 1:** Complete composition of Zarrouk medium.

Macronutrients	Final Composition (g L^−1^)
NaHCO_3_ ^1^	16.8
K_2_HPO_4_	0.5
NaNO_3_	2.5
K_2_SO_4_	1.0
NaCl	1.0
MgSO_4_·7H_2_O	0.2
CaCl_2_	0.04
FeSO_4_·7H_2_O	0.01
EDTA	0.08
**Micronutrients Solutions**	**Final Composition (mL L^−1^)**
A5 ^2^	1.0
B6 ^2^	1.0

Notes: ^1^ Present only in the autotrophic control media (PCL and NCL). The heterotrophic treatments were prepared without NaHCO_3_, while the autotrophic controls retained the original Zarrouk formulation. ^2^ The compositions of the A5 and B6 stock solutions are presented in Appendix A (Table A1).

**Table 2:** Maximum biomass concentration (X_max_) and maximum biomass productivity (P_max_) of *Spirulina* sp. LEB 18 cultures under autotrophic and heterotrophic conditions.

Conditions	Parameters	
	X_max_ (g L^−1^)	P_max_ (mg L^−1^ d^−1^)
NCL	2.53 ^b^ ± 0.12	386.92 ^a,b^ ± 106.49
PCL	3.39 ^a^ ± 0.16	447.76 ^a^ ± 75,27
0.2 G	0.44 ^d^ ± 0.07	160.13 ^c^ ± 58.90
1.0 G	0.53 ^c,d^ ±0.04	201.79 ^c^ ± 65.45
2.0 G	0.66 ^c^ ± 0.09	234.18 ^b,c^ ± 58.90
0.2 NaAc	0.63 ^c^ ≤ 0.01	252.7 ^b,c^ ± 33.01
1.0 NaAc	0.61 ^c^ ± 0.07	312.86 ^b^ ± 39.27
2.0 NaAc	0.61 ^c^ ± 0.02	303.6 ^b^ ± 26.18

Notes: (NCL) Negative Control Light, (PCL) Positive Control Light, (0.2 G) 0.2 g L^−1^ Glucose, (1.0 G) 1.0 g L^−1^ Glucose, (2.0 G) 2.0 g L^−1^ Glucose, (0.2 NaAc) 0.2 g L^−1^ Sodium Acetate, (1.0 NaAc) 1.0 g L^−1^ Sodium Acetate and (2.0 NaAc) 2.0 g L^−1^ Sodium Acetate. Different lowercase letters in the same column indicate significant differences (*p* < 0.05) for the response.

**Table 3:** Concentrations of carbohydrates, proteins, and lipids in the biomass of *Spirulina* sp. LEB 18 grown under autotrophic light/dark photoperiod (12/12 h) and heterotrophic conditions.

Conditions	Macromolecules (wt%)		
	Protein	Carbohydrates	Lipids
NCL	16.68 ^e^ ± 0.99	17.46 ^a^ ± 0.90	6.83 ^b^ ± 0.43
PCL	38.68 ^a^ ± 2.44	17.36 ^a^ ± 1.01	7.48 ^b^ ± 0.56
0.2 G	20.38 ^d,e^ ± 0.76	5.42 ^c,e^ ± 0.24	13.04 ^a^ ± 1.28
1.0 G	23.38 ^c,d^ ± 1.37	6.14 ^c,d^ ± 0.12	6.21 ^b^ ± 0.48
2.0 G	20.21 ^d,e^ ± 0.10	14.47 ^b^ ± 1.16	5.31 ^b^ ± 0.50
0.2 NaAc	32.18 ^a,b^ ± 1.33	8.33 ^d^ ± 0.48	6.96 ^b^ ± 0.17
1.0 NaAc	20.49 ^d^ ± 1.96	3.06 ^e,f^ ± 0.29	6.75 ^b^ ± 0.24
2.0 NaAc	26.87 ^b,c^ ± 0.23	1.16 ^f^ ± 0.02	10.97 ^a^ ± 0.13

Notes: (NCL) Negative Control Light, (PCL) Positive Control Light, (0.2 G) 0.2 g L^−1^ Glucose, (1.0 G) 1.0 g L^−1^ Glucose, (2.0 G) 2.0 g L^−1^ Glucose, (0.2 NaAc) 0.2 g L^−1^ Sodium Acetate, (1.0 NaAc) 1.0 g L^−1^ Sodium Acetate and (2.0 NaAc) 2.0 g L^−1^ Sodium Acetate. Different lowercase letters in the same column indicate significant differences (*p* < 0.05) for the same response.

**Table 4:** Color Perception of Different Biomass Obtained from *Spirulina* sp. LEB 18.

Conditions	L*	a*	b*
NCL	36.71 ^c^ ± 0.63	−5.74 ^b^ ± 0.15	6.99 ^d^ ± 0.12
PCL	37.95 ^c^ ± 2.02	−7.16 ^a^ ± 0.31	5.21 ^c^ ± 0.20
0.2 G	42.71 ^a,b,c^ ± 4.59	−3.28 ^d^ ± 0.36	3.54 ^e^ ± 0.36
1.0 G	47.37 ^a,b^ ± 2.00	−2.14 ^e^ ± 0.10	4.87 ^c^ ± 0.21
2.0 G	42.82 ^a,b,c^ ± 1.80	−3.78 ^c,d^ ± 0.16	2.88 ^e^ ± 0.13
0.2 NaAc	43.15 ^a,b,c^ ± 3.57	−7.75 ^a^ ± 0.55	14.78 ^a^ ± 1.00
1.0 NaAc	41.76 ^b,c^ ± 2.19	−5.74 ^b^ ± 0.28	15.04 ^a^ ± 0.61
2.0 NaAc	49.12 ^a^ ± 0.81	−4.23 ^c^ ± 0.10	9.84 ^b^ ± 0.18

Notes: (PCL) Positive Control Light, (NCL) Negative Control Light, (0.2 G) 0.2 g L^−1^ Glucose, (1.0 G) 1.0 g L^−1^ Glucose, (2.0 G) 2.0 g L^−1^ Glucose, (0.2 NaAc) 0.2 g L^−1^ Sodium Acetate, (1.0 NaAc) 1.0 g L^−1^ Sodium Acetate and (2.0 NaAc) 2.0 g L^−1^ Sodium Acetate. Different lowercase letters in the same column indicate significant differences (*p* < 0.05) for the same response.

**Table 5:** Chroma (C*ab), Hue Angle (h°ab), and Color Difference (ΔE*) of biomass obtained from *Spirulina* sp. LEB 18.

Conditions	C*ab	h°ab	ΔE*
PCL	8.86 ^c^ ± 0.37	−0.63 ^g^ ± 0.01	0
NCL	9.04 ^c^ ± 0.11	−0.88 ^d^ ± 0.02	3.06 ± 0.95
0.2 G	4.82 ^d^ ± 0.50	−0.82 ^e^ ± 0.01	7.79 ± 3.58
1.0 G	5.32 ^d^ ± 0.23	−1.16 ^b^ ≤ 0.01	11.46 ± 1.26
2.0 G	4.76 ^d^ ± 0.20	−0.65 ^f^ ≤ 0.01	7.67 ± 1.09
0.2 NaAc	16.69 ^a^ ± 1.14	−1.09 ^c^ ≤ 0.01	10.33 ± 3.19
1.0 NaAc	16.10 ^a^ ± 0.66	−1.21 ^a^ ≤ 0.01	9.58 ± 1.39
2.0 NaAc	10.71 ^b^ ± 0.20	−1.16 ^b^ ≤ 0.01	12.83 ± 1.35

Notes: (PCL) Positive Control Light, (NCL) Negative Control Light, (0.2 G) 0.2 g L^−1^ Glucose, (1.0 G) 1.0 g L^−1^ Glucose, (2.0 G) 2.0 g L^−1^ Glucose, (0.2 NaAc) 0.2 g L^−1^ Sodium Acetate, (1.0 NaAc) 1.0 g L^−1^ Sodium Acetate and (2.0 NaAc) 2.0 g L^−1^ Sodium Acetate. Different lowercase letters in the same column indicate significant differences (*p* < 0.05) for the same response.

## Data Availability

The original contributions presented in the study are included in the article, further inquiries can be directed to the corresponding author.

## Appendix A

The trace metal solution (micronutrients) used in the preparation of Zarrouk medium [30] is detailed in Table A1.

**Table A1:** Compositions of A5 and B6 solutions.

A5 Solution	
Components	Concentration (g L^−1^)
H_3_BO_3_	2.86
MnCl_2_·4H_2_O	1.81
ZnSO_4_·7H_2_O	0.222
CuSO_4_·5H_2_O	0.079
NaMoO_4_	0.015
**B6 Solution**	
Components	Concentration (g L^−1^)
NH_4_VO_3_	0.023
K_2_Cr_2_(SO_4_)_4_·24H_2_O	0.096
NaWO_4_·2H_2_O	0.018
TiOSO_4_·H_2_SO_4_·8H_2_O	0.0611
Co(NO_3_)_2_·6H_2_O	0.044
